# Mosquito larvicidal activities of *Solanum villosum *berry extract against the dengue vector *Stegomyia aegypti*

**DOI:** 10.1186/1472-6882-8-10

**Published:** 2008-04-03

**Authors:** Nandita Chowdhury, Anupam Ghosh, Goutam Chandra

**Affiliations:** 1 Mosquito and Microbiology Research Units, Parasitology Laboratory, Department of Zoology, Burdwan University, West Bengal, India

## Abstract

**Background:**

Vector control is facing a threat due to the emergence of resistance to synthetic insecticides. Insecticides of botanical origin may serve as suitable alternative biocontrol techniques in the future. Although several plants have been reported for mosquitocidal activity, only a few botanicals have moved from the laboratory to field use, because they are poorly characterized, in most cases active principals are not determined and most of the works are restricted to preliminary screening. *Solanum villosum *is a common weed distributed in many parts of India with medicinal properties, but the larvicidal activity of this plant has not been reported so far.

**Methods:**

Aqueous and polar/non-polar solvent extract of fresh, mature, green berries of *S. villosum *was tested against *Stegomyia aegypti*, a common vector of dengue fever. A phytochemical analysis of chloroform:methanol extract was performed to search for the active toxic ingredient. The lethal concentration was determined (log probit analysis) and compared with Malathion. The chemical nature of the active substance was also evaluated following ultraviolet-visual (UV-Vis) and infrared (IR) analysis.

**Results:**

In a 72 hour bioassay experiment with the aqueous extract, the highest mortality was recorded in 0.5% extract. When the mortality of different solvent extracts was compared, the maximum (*p *< 0.05) mortality was recorded at a concentration of 50 ppm of chloroform:methanol extract (1:1, v/v). The larvicidal activity was lower when compared with the chemical insecticide, Malathion (*p *< 0.05). Results of regression analysis revealed that the mortality rate (*Y*) was positively correlated with the period of exposure (*X*) and the log probit analysis (95% confidence level) recorded lowest value (5.97 ppm) at 72 hours of exposure. Phytochemical analysis of the chlororm:methanol extract reported the presence of many bioactive phytochemicals. Two toxic compounds were detected having *R*_f _= 0.82 (70% and 73.33% mortality in 24 and 48 hours, respectively) and *R*_f _= 0.95 (40% and 50% mortality in 24 and 48 hours, respectively). IR analysis provided preliminary information about the steroidal nature of the active ingredient.

**Conclusion:**

*S. villosum *offers promise as potential bio control agent against *S. aegypti *particularly in its markedly larvicidal effect. The extract or isolated bioactive phytochemical could be used in stagnant water bodies for the control of mosquitoes acting as vector for many communicable diseases.

## Background

Mosquitoes transmit several public health problems, such as malaria, filariasis, dengue and Japanese encephalitis, causing millions of deaths every year [1]. *Stegomyia aegypti *(= *Aedes aegypti*) is a vector for an arbovirus responsible for dengue fever, dengue haemorrhagic fever and dengue shock syndrome, and with unusual manifestations such as central nervous system involvement [2,3]. About two-fifths of the world's populations are at risk of catching dengue [4-6].

Mosquitoes in the larval stage are attractive targets for pesticides because they breed in water and, thus, are easy to deal with them in this habitat. The use of conventional chemical pesticides has resulted in the development of resistance [7,8], undesirable effects on non-target organisms and fostered environmental and human health concerns [9]. The use of herbal products is one of the best alternatives for mosquito control. The search for herbal preparations that do not produce any adverse effects in the non-target organisms and are easily biodegradable remains a top research issue for scientists associated with alternative vector control [10].

*Solanum villosum *(Solanaceae: Solanales), commonly known as red-fruit nightshade, is widely distributed in many parts of India. This is an Ayurvedic herb with multiple medicinal properties [11]. The objective of the present study was to examine the larvicidal activity of aqueous, polar and non-polar solvent extracts of the green berries of this plant against the larvae of *S. aegypti *mosquitoes and to gather preliminary information about the nature of the active ingredient responsible for larval mortality.

## Methods

### Test mosquitoes

The present study was conducted at Burdwan (23° 16' N, 87° 54' E), West Bengal, India, during June-August 2006. Larvae of *S. aegypti *were obtained from a laboratory colony maintained in the Mosquito Research Unit, Department of Zoology, The University of Burdwan. The colony was kept free from exposure to pathogens, insecticides or repellents and maintained at 25–30°C. The larvae were fed on a powdered mixture of dog biscuits and dried yeast powder at a ratio of 3:1. The adult colony was provided with 10% sucrose solution and 10% multivitamin syrup, and was periodically blood-fed on restrained rats.

### Preparation of aqueous extracts

Fresh, mature, green berries of *S. villosum *were randomly harvested during the study period from plants growing on the outskirts of Burdwan. All the berries were initially rinsed with distilled water and dried on a paper towel. The crude extracts were prepared by grinding the plant material in a mortar and pestle and passing the ground material through Whatman No 1 filter paper. Required concentrations of aqueous extracts were prepared by mixing the crude extract with a suitable amount of sterilized distilled water.

### Preparation of plant extracts in different solvent systems

We harvested 25 g of fresh, mature berries, which were rinsed with distilled water and dried in a shed. The dried berries were put in a Soxhlet apparatus and the plant extracts were prepared using five solvents, namely petroleum ether, benzene, chloroform:methanol (1:1, v/v), acetone and absolute alcohol, applying one after another (extraction period 72 hour for each solvent and the temperature was < 40°C). The extracts were collected separately and the column of the Soxhlet apparatus was washed with 200 ml of water and 100 ml of a similar solvent as an eluent after each type of solvent extraction procedure. The eluted materials and each type of extract were concentrated in combination at 40°C to 100 ml of extract by evaporation in a rotary evaporator. Then each of the extracts was filtered, solvents were evaporated and the solid residues were weighed and then dissolved in a suitable amount of sterilized distilled water for the formulation of graded concentrations. The total yield of each extract from 25 g of berries was as follows: petroleum ether extract, 1.26 g; benzene extract, 2.38 g; chloroform:methanol (1:1, v/v) extract, 4.33 g; acetone extract, 3.00 g; and absolute alcohol extract 2.36 g.

### Larvicidal bioassay

The larvicidal bioassay followed the World Health Organization (WHO) standard protocols [12] with slight modifications. Each of the concentrations of aqueous berry extract (0.1–0.5%) was transferred into sterile glass Petri dishes (9 cm diameter/150 ml capacity). Ten third instar larval form of *S. aegypti *were separately introduced into different Petri dishes containing graded concentrations and the mortality were recorded after 24, 48 and 72 hours of the exposure period. The data of mortality in 48 and 72 hours were expressed by the addition of the mortality at 24 and 48 hours, respectively. Dead larvae were identified when they failed to move after probing with a needle in the siphon or cervical region. The experiments were replicated three times and conducted under laboratory conditions at 25–30°C and 80–90% relative humidity. Similar types of bioassay were conducted with different polar and non-polar solvent extracts (concentrations of 50, 25 and 15 ppm) of the green berries and with a chemical insecticide, Malathion, on third instar larval forms, and chloroform:methanol (1:1, v/v) extract on first and fourth instar larval forms. Larval toxicity was also tested according to similar methodologies using the bioactive substances (from chloroform:methanol extract) isolated from thin-layer chromatographic (TLC) plates.

### Phytochemical analysis

The phytochemical analysis was carried out using the chloroform:methanol extract (as it exhibited highest mortality against *S. aegypti *larvae) of the green berries of *S. villosum *using the standard methods of Harbone [13] and Stahl [14]. One or two drops of the chloroform:methanol extract were applied (using a capillary tube) to the bottom of each of the pre-coated and pre-heated (100°C for 30 minutes) glass plates (eight glass plates), which were prepared with silicagel G using Unoplan coating apparatus (Shadon, London). After 5 minutes of drying, each of the plates was placed in the separate glass chamber for TLC analysis, with different solvent systems as the mobile phase. After the movement of solvent at the top of the plates, each plate was removed from the glass chamber and separately air-dried. After 10 minutes each of plates was sprayed with a different spraying reagent for the identification of appropriate phytochemical. The phytochemicals included in the study were sapogenins, steroid, terpenoids, flavonoids, alkaloid, essential oils, phenolics and amino acids. A qualitative test was carried out to indicate the presence of saponins [15]; the remaining phytochemicals were determined using TLC analysis by the application of suitable solvents and spray reagents and, in each case, *R*_f _values were recorded.

### Ultraviolet-visual and infrared analysis of the active ingredient

The chloroform:methanol extract of the green berries of *S. villosum *was further chromatogrammed (30 plates) without the application of spraying reagents and each of the spots showed positive activity were separately scrapped according to their respective R_f _values. Then each of the spots with their distinguishing R_f _value was combined (from 30 plates) and undergoes further bioassay experiment to reveal the nature of active ingredient. As the spots exhibited positive response in Liberman Buchard reagent recorded highest larval mortality during further bioassay experiments, it undergoes spectral analysis by ultraviolet-visual (UV-Vis) and infrared (IR) spectroscopy. The UV-Vis analysis was carried out using a UV-1601 PC, SHIMADZU spectrophotometer with medium scan speed and sampling interval of 0.5 seconds. The IR spectroscopy analysis of the active spot was performed using KBr plates (JASCO FT-IR Model-420) with a scanning speed of 2 mm s^-1^.

All solvents and reagents used were of analytical grade and purchased from E. Merck, India. The TLC silica gel plates (0.25 mm thickness) were prepared and equilibrated with 2% (w/w) of water before use.

### Statistical analysis

The percentage mortality observed (%M) was corrected using Abbott's formula [16] during the observation of the larvicidal potentiality of the plant extracts. A Student's *t*-test was performed to find the significance between the concentration of plant extract and mortality at different periods with different instars. Statistical analysis of the experimental data was performed using the computer software Statplus 2006 and MS EXCEL 2002 to find the LC_50_, regression equations (*Y *= mortality; *X *= concentrations) and regression coefficient values.

## Results

The results of the present study indicate that the mortality rates at a 0.5% concentration were highest amongst all concentrations of the aqueous extracts tested for larval mortality and it was significantly higher (*p *< 0.05) than the mortality rates at 0.1%, 0.2%, 0.3% and 0.4% concentration of aqueous plant extract at 24, 48 and 72 hours of exposure (Table 1). The mortality of the same instar larval form with different polar and non-polar solvent extracts is presented in Table 2. The larvicidal potentiality of chloroform:methanol (1:1, v/v) extract was further tested for first and fourth instar larvae: the highest mortality was recorded at 15 ppm for first instar larvae and it is statistically more significant (*p *< 0.05) than both the 5 and 10 ppm concentrations and at both 48 and 72 hours of exposure (Table 3).

**Table 1:** The larvicidal activity (mean mortality ± standard error) of different concentrations of aqueous extract of the green berries of *S. villosum *on third instar larvae of *S. aegypti*. Student's *t*-test *t *= 29.42*, 5.5*, 17.0* (between 0.5% and 0.1%) 12.43*, 3.32*, 14.0* (between 0.5% and 0.2%) and 1.73*, 4.33*, 4.0* (between mortality in 0.5% and 0.3% plant extract at 24, 48 and 72 hours bioassay); * denotes significant (*p *< 0.05); table value = 2.92 at five degrees of freedom. M, mortality (%); SE, standard error.

	**Period of exposure (hours)**		
	
**Concentration (%)**	**24**	**48**	**72**
0.1	20 ± 5.77	26.67 ± 8.67	30 ± 8.81
0.2	30 ± 7.69	36.67 ± 5.77	40 ± 7.69
0.3	60 ± 5.57	70 ± 1.92	73.33 ± 3.84
0.4	66.66 ± 1.92	70 ± 1.92	76.66 ± 5.57
0.5	76.66 ± 1.92	86.66 ± 5.77	90 ± 1.92

**Table 2:** Efficacy of different concentrations of polar and non-polar solvent extracts of the green berries of *S. villosum *on third instar larvae of *S. aegypti*. M, mortality (%); S, survivality (%).

**Type of solvents**	**Concentrations (ppm)**	**Period of exposure (hours)**					
		
		**24**		**48**		**72**	
		
		**M**	**S**	**M**	**S**	**M**	**S**
Petroleum ether	50	3.33	96.67	6.67	93.33	13.33	86.67
	25	3.33	96.67	3.33	96.67	10	90
	15	0	100	3.33	96.67	6.67	93.33
Benzene	50	6.67	93.33	13.33	86.67	23.33	76.67
	25	3.33	96.67	6.67	93.33	16.67	83.33
	15	3.33	96.67	6.67	93.33	13.33	86.67
Chloroform: methanol	50	70	30	73.33	26.66	76.66	23.33
	25	53.33	46.66	56.66	43.33	56.66	43.33
	15	40	60	43.33	56.66	43.33	56.66
Acetone	50	6.67	93.33	10	90	13.33	86.67
	25	6.67	93.33	10	90	10	90
	15	3.33	96.67	6.67	93.33	10	90
Absolute alcohol	50	30	70	36.67	63.63	50	50
	25	13.33	86.67	23.33	76.67	33.33	66.67
	15	10	90	16.67	83.33	26.67	73.33

**Table 3:** The larvicidal potentiality (mean mortality ± standard error) of different concentrations of chloroform:methanol (1:1, v/v) extract of the green berries of *S. villosum *and a synthetic insecticide, Malathion, on first and fourth instars larvae of *S. aegypti*. For first instar larvae: *t *= 2.07^NS^, 3.14*, 7.56* (between 15 and 10 ppm at 24, 48 and 72 hours); *t *= 5.2*, 26.62*, 13.99* (between 15 and 5 ppm at 24, 48 and 72 hours). For fourth instar larvae: *t *= 2^NS^, 1.99^NS^, 0.91^NS ^(between 15 and 10 ppm at 24, 48 and 72 hours); *t *= 1.73^NS^, 1.89^NS^, 1.82^NS ^(between 15 and 5 ppm at 24, 48 and 72 hours). * denotes significant (*p *< 0.05); NS, not significant (*p *> 0.05). Table value = 2.92 at five degrees of freedom. M, mortality (%); SE, standard error.

**Instars of mosquito larvae**	**Concentrations (ppm)**	**Period of exposure (hours)**		
		
		**24**	**48**	**72**
First	15	60 ± 1.92	66.67 ± 5.67	70 ± 2.93
	10	40 ± 5.57	43.33 ± 3.84	46.66 ± 3.84
	5	40 ± 5.57	40 ± 5.67	53.33 ± 1.92
	Malathion (5 ppm)	100 ± 0.00	100 ± 0.00	100 ± 0.00

Fourth	15	40 ± 1.92	43.33 ± 3.84	46.66 ± 5.57
	10	33.33 ± 3.84	40 ± 1.92	43.33 ± 5.56
	5	30 ± 5.57	33.33 ± 2.93	36.67 ± 2.84
	Malathion (5 ppm)	100 ± 0.00	100 ± 0.00	100 ± 0.00

**Figure 1:**
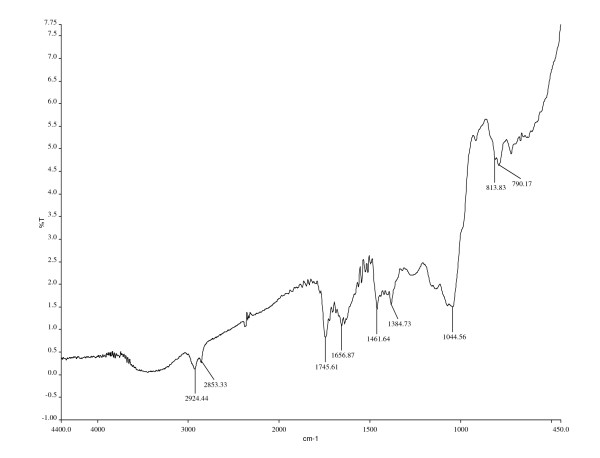
**Interpretation of IR spectra of the compound having *R*_f _= 0.95**. Frequency range and probable functional groups of the compound (*R*_f _= 0.946): 2,924.44 and 2,853.33 cm^-1^, C-H (S) group; 1,745.61 cm^-1^, C = O (S) stretch; 1,656.87 cm^-1^, asymmetrical stretch of NO_2_ compounds (S); 1,461.64 cm^-1^, scissoring and bending of C-H compounds (V); 1,384.73 cm^-1^, symmetrical stretches of NO2B _B_compounds (S); 1,044.56 cm^-1^, C-O stretch (S); 813.83 cm^-1^, phenyl ring substitution bands (S); 790.17 cm^-1^, C-H bend (B). V, variable; M, medium; S, strong; Br, broad; W, weak.

**Figure 2:**
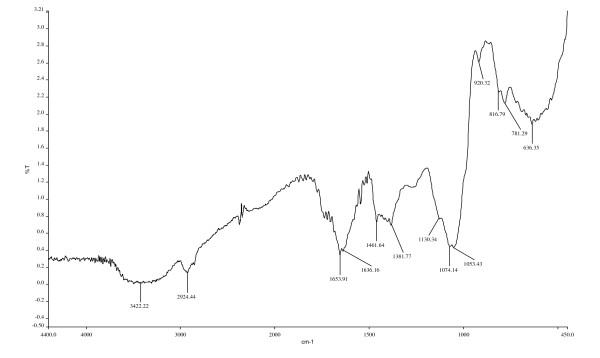
**Interpretation of IR spectra of the compound having *R*_f _= 0.82**. Frequency range and probable functional groups of the compound (*R*_f _= 0.818): 3,422.22 cm^-1^, H bonded OH stress (B); 2,924.44 cm^-1^, CH (S) stretch; 1,653.91 cm^-1^, asymmetrical stretch of NO_2_ compounds (S); 1,636.16 cm^-1^, NH (M) bond; 1,461.64 cm^-1^, scissoring and bending of C-H compounds (V); 1,381.77 cm^-1^, doublet isopropyl (M-W); 1,130.34, 1,074.14, 1,053.43 cm^-1^, CO group (S) stretch; 920.32 cm^-1^, alkenes (S) bend; 816.79, 781.29 cm^-1^, CH phenyl ring (S) substitution bend; 636.35 cm^-1^, alkynes bend (B). V, variable; M, medium; S, strong; Br, broad; W, weak.

However, no significant difference was recorded for fourth instar larvae between 15 and 10 ppm concentrations and 15 and 5 ppm concentrations. An absolute mortality (100%) was observed within 24 hours during the exposure to the chemical insecticide, Malathion (5 ppm concentration). The results of regression analysis revealed that the mortality rate (*Y*) is positively correlated with the period of exposure (*X*) having a regression coefficient close to one in each case (Table 4). The results of log probit analysis (95% confidence level) revealed that LC_50 _values gradually decreased with the exposure periods having the lowest value at 72 hours of exposure to third instar larvae, followed by first and fourth instar larvae. The results of preliminary phytochemical analysis of the chloroform:methanol extract of the green berries of *S. villosum *are presented in Table 5. A qualitative test indicated the presence of saponins and chromatographic analysis revealed the presence of steroids, alkaloids, terpenoids, saponins, amino acids, phenolics, flavonoids and essential oil as major phytochemicals and the absence of the sapogenins following the application of different solvent systems and spraying reagents. When the isolated compounds from the TLC plates were further bio-assayed against the third instar larvae, the mortality was recorded in two compounds. The highest mortality (at a concentration of 50 ppm) was recorded in the first compound having *R*_f _= 0.818 (70% and 73.33% in 24 and 48 hours, respectively) followed by a second compound having *R*_f _= 0.946 (40% and 50% in 24 and 48 hours, respectively) with maximum absorption at 297.50 and 361.00 nm, respectively, during UV-Vis analysis. IR analysis of two compounds and their respective functional groups are shown in Figures 1 and 2.

**Table 4:** Log probit analysis of the larvicidal activity of chloroform:methanol extract of the green berries of *S. villosum *on different instar larvae of *S. aegypti*. LC, lethal concentration; *R*, coefficient of regression equations.

**Type of instars of mosquito larvae**	**Period of bioassay (hours)**	**Regression equations**	***R*^2^**	**LC_50 _values (ppm)**	**Lower and upper fiducidal limits (ppm)**
First	24	*Y *= 29.82 + 0.820*x*	0.97	22.06	16.05–27.66
	48	*Y *= 33.16 + 0.820*x*	0.97	19.58	14.36–24.20
	72	*Y *= 31.19 + 0.923*x*	0.98	19.19	13.97–23.45

Third	24	*Y *= 29.83 + 0.820*x*	0.97	11.67	8.49–14.84
	48	*Y *= 33.16 + 0.820*x*	0.97	9.54	6.82–12.25
	72	*Y *= 31.19 + 0.923*x*	0.98	5.97	2.15–9.79

Fourth	24	*Y *= 25.98 + 0.282*x*	0.99	49.84	44.53–54.77
	48	*Y *= 31.19 + 0.256*x*	0.82	21.22	13.30–29.13
	72	*Y *= 34.53 + 0.256*x*	0.82	21.02	15.97–73.94

**Table 5:** Phytochemical analysis of the chloroform:methanol extract of the green berries of *S. villosum*

**Solvents used**	**Spraying reagents**	***R*_f _values (and appearance) of the positive spot**	**Presence/absence of phytochemicals**
Acetone-hexane (4:1)	Antimony chloride in concentrated hydrochloric acid	-	Absence of sapogenins
Methanol-concentrated ammonium hydroxide (200:3)	Dragendorff	0.95, 0.96 (green)	Presence of alkaloids
Chloroform	Libermann-Buchard	0.95, 0.82, 0.68 (reddish pink)	Presence of steroids
Chloroform:benzene (1:1)	Vanillin-sulphuric acid	0.99 (violet blue)	Presence of essential oil
Chloroform:acetic acid:water (90:45:6)	Saturated alcoholic sodium acetate	0.98 (green)	Presence of flavonoids
Ethyl acetate:benzene (1:1)	Folin reagent	0.98, 0.94 (blue)	Presence of phenolics
	
	Ninhydrin	0.78	Presence of protein
Chloroform on silica gel plate treated with silver nitrate	Antimony chloride in chloroform	0.97, 0.48 (green)	Presence of terpenoids

## Discussion

Nowadays, mosquito control is mostly directed against larvae and only against adults when necessary. This is because the fight against adult is temporary, unsatisfactory and polluting for the environment, while larval treatment is more localized in time and space resulting in less-dangerous outcomes. Larval control can be an effective control tool due to the low mobility of larval mosquitoes, especially where the principal breeding habitats are man-made and can be easily identified [17].

The secondary compounds of plants make up a vast repository of compounds with a wide range of biological activities. Most studies report active compounds as steroidal saponins. Saponins are freely soluble in both organic solvents and water, and they work by interacting with the cuticle membrane of the larvae, ultimately disarranging the membrane, which is the most probable reason for larval death [18]. Wiesman and Chapagain [19] reported that saponin extracted from the fruit of *Balanites aegyptica *showed 100% mortality against larvae of *S. aegypti*. The larvicidal property of a saponin mixture isolated from *Cestrum diurnum *was also evaluated against *Anopheles stephensi *mosquito by Ghosh and Chandra [20]. The impact of phenolic compounds on the mosquito larvae has also been reported by many authors [21,22]. Aluminium chloride obtained from alder leaf, known for its phenolic complexing activity, is also reported to have the larvicidal activity against *S. aegypti *[23]. Isoflavonoids from tubers of *Neorautanenia mitis *had a larvicidal effect against the malaria and filariasis transmitting mosquitoes, *Anopheles gambiae *and *Cx. quinquefaciatus*, respectively [24]. Essential oils extracted from Brazilian plants exhibited larvicidal activity against *S. aegypti*, with LC_50 _values ranging from 60 to 538 ppm (see [25]). Studies with *Lippia sidoides *[26] and *Cymbopogon citrates *[27] essentials oils suggested that they are a promising biocontrol agent against *S. aegypti*. Rohini et al [28] isolated D-pinitol, from the EtOH extract of *Acacia nilotica*, which showed larvicidal activity. Alkaloids derived from *Piper longum *fruit [29] and *Triphyophyllum pellatum *[30] showed larvicidal activity against *C. pipiens and A. stephensi*, respectively. Khanna and Kannabiran [31] reported the role of tannin compounds extracted from *Hemidesmus indicus*, *Gymnema sylvestre *and *Eclipta prostrate *that causes mortality in *Cx*. *quinquefasciatus *larvae.

The present study indicates that green berries of *S. villosum *had biocontrol activity against *S. aegypti*. The highest mosquitocidal activity was noted in chloroform:methanol extract. The qualitative and chromatographic study of green berries of *S. villosum *revealed the presence of several bioactive compounds. However, the IR spectra of the bioactive compounds during the present study also indicated that any steroid compound(s) is responsible for larval toxicity.

## Conclusion

In conclusion, *S. villosum *offers promised as a potential bio control agent against *S. aegypti *particularly in its markedly larvicidal effect. The biocontrol potentiality was lower than chemical insecticides such as Malathion. The extract or isolated bioactive phytochemical from the plant could be used in stagnant water bodies which are known to be the breeding grounds for mosquitoes. However, further studies on the identification of the active principals involved and their mode of action and field trials are needed to recommend *S. villosum *as an anti-mosquito product used to combat and protect from mosquitoes in a control program.

## Competing interests

The author(s) declare that they have no competing interests.

## Authors' contributions

NC carried out the laboratory bioassay experimentation and phytochemical analysis of the extract. AG participated in the statistical and spectroscopic analysis and drafted the manuscript. GC participated in the conception, design of experiments, critical revision of the manuscript and coordination. All authors read and approved the final manuscript.

## Pre-publication history

The pre-publication history for this paper can be accessed here:


